# Prevalence of functional bowel disorders and faecal incontinence: an Australian primary care survey

**DOI:** 10.1111/codi.12808

**Published:** 2015-01-21

**Authors:** K.‐S. Ng, N. Nassar, K. Hamd, A. Nagarajah, M. A. Gladman

**Affiliations:** ^1^ Academic Colorectal Unit Sydney Medical School – Concord University of Sydney Sydney New South Wales Australia; ^2^ Clinical and Population Perinatal Health Research Kolling Institute of Medical Research University of Sydney Sydney New South Wales Australia; ^3^ School of Medicine University of Western Sydney Sydney New South Wales Australia

**Keywords:** Functional bowel disorders, faecal incontinence, pelvic floor disorders, epidemiology, health‐service provision

## Abstract

**Aim:**

Interest in functional bowel disorders (FBDs) and faecal incontinence (FI) has increased amongst coloproctologists. The study aimed to assess the prevalence of FBDs and FI (including its severity) among Australian primary healthcare seekers using objective criteria.

**Method:**

A cross‐sectional survey was conducted in a primary care setting in Sydney, Australia. A self‐administered questionnaire was used to collect demographic information and diagnose FBDs (irritable bowel syndrome, constipation, functional bloating and functional diarrhoea) based on Rome III criteria. The severity of FI was determined using the Vaizey incontinence score. Associations with medical/surgical history and healthcare utilization were assessed.

**Results:**

Of 596 subjects approached, 396 (66.4%) agreed to participate. Overall, 33% had FBD and/or FI. Irritable bowel syndrome was present in 11.1% and these participants were more likely to report anxiety/depression (*P *<* *0.01) and to have had a previous colonoscopy (*P *<* *0.001) or cholecystectomy (*P *=* *0.02). Functional constipation was present in 8.1%, and functional bloating and functional diarrhoea were diagnosed in 6.1%, and 1.5%, respectively. FI was present in 12.1% with the majority (52%) reporting moderate/severe incontinence (Vaizey score > 8). Participants with FI were more likely to have irritable bowel syndrome, urinary incontinence and previous anal surgery (*P *<* *0.01).

**Conclusion:**

FBDs and FI are prevalent conditions amongst primary healthcare seekers and the needs of those affected appear to be complex given their coexisting symptoms and conditions. Currently, the majority do not reach colorectal services, although increased awareness by primary care providers could lead to sufferers being referred for specialist management.

**What does this paper add to the literature?**

This paper is the first to establish the prevalence of functional bowel disorders and faecal incontinence using explicit, standardized criteria amongst healthcare seekers who have the potential to access colorectal services, rather than in the community. Further, it has identified important associations of these disorders.

## Introduction

Interest in the management of pelvic floor and functional gastrointestinal disorders (FGIDs) has increased dramatically amongst colorectal surgeons in the last decade, reflecting the expanding spectrum of non‐surgical 1 and surgical therapeutic options 2, 3. Indeed, the management of these patients now forms an important part of the work of the coloproctologist and is no longer confined to a few specialist centres. FGIDs represent various combinations of chronic or recurrent gastrointestinal symptoms that are not attributable to gross structural or biochemical abnormalities 4, 5, 6 and thus, in the absence of objective biomarkers, their diagnosis has relied heavily on the development of symptom‐based criteria. The most widely accepted are the Rome criteria, which have provided a structured and comprehensive classification system of FGIDs 4, 7. Despite the limitations of a purely symptom‐based taxonomy 8, the Rome criteria are now published in their third version 9 and are widely employed to provide consistency in the classification of subjects in the research setting. Further, such a taxonomy is advantageous from a clinical perspective, since a diagnosis is reached without the need for invasive investigation 7.

From the perspective of the colorectal surgeon, FGIDs attributed to dysfunction of the lower gastrointestinal tract include the functional bowel disorders (FBDs) [including irritable bowel syndrome (IBS), functional constipation, functional bloating and functional diarrhoea] and faecal incontinence (FI). IBS is characterized by abdominal pain or discomfort associated with change in bowel frequency and/or consistency 10 and is the most commonly diagnosed functional disorder 11, 12, 13, accounting for up to 36% of all visits to gastroenterologists 14, 15. Functional constipation is defined as persistently difficult, infrequent or seemingly incomplete defaecation 10, and was found to affect up to 27% of North Americans in a recent systematic review 16. Functional bloating, defined as a recurrent sensation of abdominal distension, appears to affect between 16% and 30% of the population 17, whilst functional diarrhoea, characterized by chronic loose/watery stools, had a prevalence of 8.5% in a recent population‐based Canadian study 18. The prevalence of FI, defined as the recurring uncontrolled passage of faecal material 19, is more difficult to measure given the reluctance of sufferers to report this symptom on account of the associated social embarrassment and stigma 20, but recent studies suggest that FI may affect up to 17% of the community 21, 22.

These estimates of prevalence suggest that FGIDs are common with perhaps as many as two‐thirds of the population experiencing at least one functional gastrointestinal symptom 5, but previous studies have used inconsistent criteria for the definition and diagnosis or have focused on individual bowel disorders in isolation. Further, all existing studies have been performed in community‐based populations and such measures may not reflect the prevalence in the health‐seeking population that can access colorectal services. By contrast, *clinic*‐based surveys offer unique information about healthcare seekers 4, potentially assisting with colorectal service planning/provision, but no such studies exist in the published literature. Therefore, the aim of the present study was to measure the prevalence of FBDs and FI (and its severity) among Australians attending primary healthcare providers using the latest Rome III criteria and an objective FI scoring system, respectively.

## Method

### Study population

A cross‐sectional survey was conducted amongst individuals attending four general practices (primary care providers) in Greater Western Sydney, Australia, between July 2011 and August 2011. The general practices were randomly selected from a prospectively maintained list of practices actively engaged in medical education and/or research. All practices were doctor‐owned bulk‐billing practices, each with over five general practitioners. Universal access to primary healthcare is available in Australia whereby patients receive free or subsidized treatment (> 85%) by doctors in general practice 23. Individuals aged 18 years or older were invited to participate on a consecutive basis as they entered into the practice waiting rooms, irrespective of their reason for attendance. Participants were advised that the study pertained to their health, but the specific topic of the study was only revealed if asked by the participant. After providing informed consent for study participation, they each completed a self‐administered questionnaire.

### Self‐administered questionnaire

The self‐administered questionnaire included Rome III diagnostic criteria for FBDs (IBS, functional constipation, functional bloating and functional diarrhoea) 10, as well as details relating to the presence and severity of symptoms of FI based on the validated modified Cleveland Clinic (Vaizey) incontinence scoring system 24. Specifically, IBS was diagnosed if recurrent abdominal pain or discomfort was experienced at least 3 days per month in the last 3 months, with overall symptom duration for at least 6 months, associated with two or more of the following: (i) improvement of abdominal pain with defaecation; (ii) onset of symptoms associated with a change in frequency of stool; (iii) onset of symptoms associated with a change in form or appearance of stool 10. Further, based on predominant stool pattern without the use of anti‐diarrhoeals or laxatives, participants diagnosed with IBS were sub‐classified as IBS with constipation (IBS‐C), IBS with diarrhoea (IBS‐D), mixed type IBS (IBS‐M) and un‐subtyped IBS (IBS‐U).

Functional constipation was diagnosed in participants who did not meet the criteria for IBS but for the last 3 months (with symptom onset at least 6 months previously) had had two or more of the following: (i) straining during at least 25% of defaecations; (ii) lumpy or hard stools in at least 25% of defaecations; (iii) sensation of incomplete evacuation for at least 25% of defaecations; (iv) sensation of anorectal obstruction or blockage for at least 25% of defaecations; (v) manual manoeuvres to facilitate at least 25% of defaecations (e.g. digital evacuation, support of the pelvic floor); or (vi) fewer than three defaecations per week 10. Functional bloating was diagnosed when there was a recurrent feeling of bloating or visible distension present for at least 3 days of the month during the last 3 months with insufficient criteria to reach a diagnosis of IBS 10, while functional diarrhoea was diagnosed when there were loose or watery stools without pain occurring in at least 75% of stools during the last 3 months 10.

Faecal incontinence was defined as leakage of solid or liquid stool (not flatus) at least once per month. When this criterion was met, the severity of the incontinence was assessed using the modified Cleveland Clinic (Vaizey) incontinence score 24. Participants were required to report frequency of incontinence to solid and liquid stools, as well as flatus, as ‘never’, ‘rarely’, ‘sometimes’, ‘weekly’ or ‘daily’. Other items assessed included (i) alteration in lifestyle, (ii) need to wear a pad or plug, (iii) need to take constipating medications and (iv) inability to defer defaecation for at least 15 min. A maximum score of 24 indicated total incontinence, whilst a score of 0 implied perfect continence. Subsequently, participants with FI were further classified on the basis of their incontinence score into those with mild (score 1–8), moderate (score 9–16) or severe (score 17–24) symptoms. Presence of coexisting urinary symptoms, particularly urinary incontinence, was also documented.

The questionnaire also recorded details relating to (a) participant demographics, including ethnicity, marital status, employment status, level of education attained, and smoking and alcohol consumption, and (b) past history, including (i) previous medical diagnoses of IBS, diabetes, mental health issues (self‐reported as anxiety, depression and/or panic attacks), (ii) previous surgical procedures and (iii) for women, previous obstetric and/or gynaecological history, including details of parity, mode of delivery and presence of perineal trauma (e.g. episiotomy, vaginal tear or instrumental delivery).

### Statistical analyses

Survey data were analysed using frequency tabulations and contingency table analyses. Where appropriate, participant characteristics were dichotomized. Association between various demographic characteristics/past histories and IBS, constipation and FI were also assessed and presented as crude odds ratios (OR) with 95% confidence intervals (95% CI). All analyses were conducted using SAS v9.2 (SAS Institute, Cary, North Carolina, USA) and *P* values < 0.05 were considered statistically significant. This study was approved by the University of Western Sydney Human Research Ethics Committee (approval number H9067).

## Results

### Response rate

Of the 596 individuals approached to complete the survey, 396 (66.4%) agreed to participate. The reasons for not doing so included time constraints (42.5%) and lack of interest (22.5%), with language barriers encountered in only 5% of participants.

### Sample characteristics

Demographic characteristics and pertinent factors in the medical history are presented in Table 1. The mean age of the sample was 47 ± 17 years, and 65% were women. The majority of respondents were Caucasian (66.3%). Most participants were married (66.3%) and employed (65.2%) and approximately half (51.9%) had completed tertiary education (either at university or vocational trade).

**Table 1:** Participant characteristics.

	Number (*n *=* *396)	Per cent
Age (years)^a^		
< 20	22	5.7
20–29	71	18.3
30–39	72	18.6
40–49	61	15.8
50–59	66	17.1
60–69	53	13.7
≥ 70	42	10.9
Men	138	35.0
Ethnicity^a^		
Caucasian	262	66.3
Asian	84	21.2
Other	49	12.4
Marital status		
Married	262	66.3
Single	70	17.7
Divorced/separated/other	63	15.9
Employment status^a^		
Employed	257	65.2
Unemployed	66	16.8
Retired	71	18.0
Highest education		
University	143	36.2
TAFE/vocational training	62	15.7
High school	178	45.1
Other	12	3.0
Alcohol (standard drinks per day)		
0	197	50.1
1	149	37.9
≥ 2	47	12.0
Body mass index^a^		
< 18.5 (underweight)	11	3.1
18.5–25.0 (normal)	130	36.4
25.0–30.0 (overweight)	123	34.5
> 30 (obese)	93	26.1
Smoking status^a^		
Current smoker	36	9.1
Ex smoker	102	25.8
Never smoked	257	65.1
Past history		
Diabetes mellitus	46	11.8
Irritable bowel syndrome	28	7.2
Neurological conditions (e.g. multiple sclerosis)	7	1.8
Mental health issues	83	21.0
Depression	56	14.4
Anxiety	52	13.3
Previous anal surgery	26	6.7
Fissure	2	0.5
Fistula	6	1.5
Haemorrhoid surgery	17	4.4
Previous abdominal procedures		
Appendicectomy	48	12.1
Cholecystectomy	23	5.8
Colonoscopy	14	3.5
Medications		
Laxatives	12	3.0
Constipating medications	35	8.9
Women (*n *=* *258)		
Parous	181	70.2
Vaginal delivery	41	22.6
Tear/episiotomy/suction/forceps^b^	97	53.6
Caesarean section^b^	43	23.8
Urinary incontinence	95	24.4

a Numbers for these variables do not add to *n *=* *396 because some participants declined to respond to certain questions.

b Had at least one delivery that was either caesarean section or tear/episiotomy.

Prevalent medical conditions reported included depression (14.4%), anxiety (13.3%) and diabetes mellitus (11.8%). A significant proportion of participants had undergone previous abdominal operations and procedures, including appendicectomy (12.1%) and cholecystectomy (5.8%). Only 3.5% of participants reported having had a previous colonoscopy, while approximately 7% of participants reported previous anal surgery, the most common being haemorrhoidal surgery. Over 70% of female participants interviewed were parous, with most reporting previous vaginal delivery and approximately half experiencing significant perineal trauma secondary to episiotomy or perineal tear(s) (see Table 1).

### Prevalence of FBD and FI and their associations

The prevalence of FBD and FI are shown in Table 2. Overall, 33% (95% CI 28–38) of all participants interviewed had either FBD and/or FI. Notably, 11% (95% CI 8–15) had IBS, 8% (95% CI 6–11) had functional constipation and 12% (95% CI 9–16) had FI.

**Table 2:** Prevalence of functional bowel disorders and faecal incontinence.

	Number	Per cent (95% CI)
Irritable bowel syndrome (IBS)	44	11.1 (8.2–14.6)
IBS‐C	11	2.8 (1.4–4.9)
IBS‐D	12	3.0 (1.6–5.2)
IBS‐M	18	4.5 (2.7–7.1)
IBS‐U	3	0.8 (0.2–2.2)
Functional constipation	32	8.1 (5.6–11.2)
Functional bloating	24	6.1 (3.9–8.9)
Functional diarrhoea	6	1.5 (0.6–3.3)
Faecal incontinence (FI)		
FI (i.e. solid + liquid stool)	48	12.1 (9.1–15.8)
Mild (Vaizey 1–8)	23	47.9
Moderate (Vaizey 9–16)	22	45.8
Severe (Vaizey 17–24)	3	6.3
Solid stool incontinence	19	4.8 (2.9–7.4)
Liquid stool incontinence	29	7.3 (5.0–10.4)
Flatus incontinence only	98	28.6 (23.5–33.2)

#### Irritable bowel syndrome

Of the sample studied, 44 (11.1%) participants fulfilled Rome III criteria for IBS, with approximately equal proportions of IBS‐C (2.8%), IBS‐D (3.0%) and IBS‐M (4.5%); only 1.5% of participants were classified as having IBS‐U (Table 2). Of the participants who met these diagnostic criteria, 70% (31/44) had *never* previously been formally diagnosed with IBS. Conversely, over half (15/28) of participants who had been given a previous ‘diagnosis’ of IBS *failed* to satisfy the objective Rome III criteria.

Irritable bowel syndrome was diagnosed twice as frequently in Caucasian participants compared with non‐Caucasian participants (OR 2.12, 95% CI 0.99–4.56). There was an increased tendency for IBS to be diagnosed amongst women, those employed, and those with tertiary or vocational education (Table 3), although these associations did not reach statistical significance. Participants with ‘self‐reported’ symptoms of mental health issues were over three times more likely to have IBS (OR 3.42, 95% CI 1.78–6.58). Participants meeting the Rome III criteria for IBS were over nine times more likely to have had a previous colonoscopy (OR 9.32, 95% CI 3.10–28.04) and over three times more likely to have had a previous cholecystectomy (OR 3.11, 95% CI 1.16–8.37). There was a tendency for increased rates of appendicectomy in IBS participants as well, although this did not reach statistical significance (Table 3).

**Table 3:** Demographic and medical associations.

	IBS		Constipation		Functional bloating		Functional diarrhoea		FI	
	OR (95% CI)	*P*	OR (95% CI)	*P*	OR (95% CI)	*P*	OR (95% CI)	*P*	OR (95% CI)	*P*
Age	N/A	0.38	N/A	0.70	N/A	0.16	N/A	***0.02***	N/A	0.09
Male gender	0.59 (0.29–1.20)	0.14	1.12 (0.53–2.37)	0.76	0.47 (0.17–1.28)	0.13	***9.59 (1.11–83.33)***	***0.01***	0.66 (0.33–1.29)	0.22
Caucasian ethnicity	2.12 (0.99–4.56)	***0.05***	1.13 (0.52–2.46)	0.76	0.84 (0.36–1.97)	0.68	2.57 (0.30–22.22)	0.37	1.42 (0.72–2.79)	0.30
Non‐married	1.28 (0.67–2.43)	0.46	0.89 (0.41–1.93)	0.76	0.64 (0.25–1.65)	0.35	a	0.08	1.48 (0.80–2.74)	0.21
Employed	1.69 (0.82–3.45)	0.15	0.58 (0.28–1.19)	0.13	1.64 (0.64–4.24)	0.30	1.07 (0.19–5.90)	0.94	0.97 (0.52–1.82)	0.92
Tertiary educated	1.72 (0.90–3.29)	0.10	1.06 (0.51–2.18)	0.88	0.65 (0.28–1.49)	0.30	1.87 (0.34–10.33)	0.47	1.01 (0.55–1.84)	0.98
≥ 2 standard alcoholic &!#6;drinks per day	0.71 (0.24–2.09)	0.53	1.41 (0.51–3.85)	0.50	1.11 (0.32–3.89)	0.87	***7.80 (1.53–39.82)***	***0.004***	1.30 (0.55–3.10)	0.55
Body mass index	N/A	0.07	N/A	0.12	N/A	0.85	N/A	***0.005***	N/A	0.24
Smoking	0.44 (0.10–1.91)	0.26	1.48 (0.49–4.48)	0.49	0.90 (0.20–4.00)	0.89	2.02 (0.23–17.80)	0.52	0.90 (0.30–2.65)	0.84
Diabetes mellitus	1.48 (0.65–3.56)	0.37	2.30 (0.92–5.62)	0.07	0.70 (0.16–3.07)	0.63	1.50 (0.17–13.15)	0.71	1.69 (0.73–3.89)	0.21
Mental health issues	***3.42 (1.78–6.58)***	***< 0.01***	1.06 (0.44–2.55)	0.89	0.99 (0.36–2.74)	0.99	a	0.20	1.87 (0.96–3.64)	0.06

N/A, not applicable; IBS, irritable bowel syndrome; FI, faecal incontinence.

a Not computed, insufficient numbers.

Bold and italics represent the significant values (*P* < 0.05).

#### Functional constipation

Rome III criteria for functional constipation were met in 32 (8.1%) participants (Table 2) of whom only one reported regular laxative consumption. No significant associations were identified between constipation and age, ethnicity or employment status (Table 3). Increased rates of diabetes, appendicectomy and cholecystectomy were also found amongst constipated participants, but these did not reach statistical significance.

#### Functional bloating

Functional bloating was diagnosed in 24 (6.1%) participants (Table 2). Whilst most cases were diagnosed in women and those younger than 60 years, associations with gender and age did not reach statistical significance. No other demographic associations were observed. Functional bloating did not appear to influence rates of colonoscopy, appendicectomy or cholecystectomy.

#### Functional diarrhoea

Criteria for functional diarrhoea were met in six (1.5%) participants (Table 2). Whilst significant associations were identified between functional diarrhoea and age greater than 60 years (OR 6.37, 95% CI 1.15–35.37), male gender (OR 9.59, 95% CI 1.11–83.33), alcohol consumption of two or more standard drinks per day (OR 7.80, 95% CI 1.53–39.82) and increased body mass index (*P *=* *0.005), these findings should be interpreted with caution given the small number diagnosed.

#### Faecal incontinence

Overall, 48 (12.1%) participants experienced FI, with incontinence to solid stool reported by 19 (39.7%) and to liquid stool (without solid stool incontinence) by the remaining 29 (60.3%). Of those diagnosed with FI, the majority (52%) were classified as having moderate or severe symptoms. Notably, a further 98 participants (28.6%) who did not fulfil the diagnostic criteria for FI reported incontinence to flatus alone (Table 2).

A significant proportion of patients experienced concomitant symptoms of IBS and FI. Specifically, one‐third (*n *=* *13) of patients with IBS had FI; put differently, IBS patients were almost four times more likely to report FI (OR 3.80, 95% CI 1.82–7.93). Previous anal surgery (for anal fissure, anal fistulae or haemorrhoids etc.) increased the risk of FI almost fourfold (OR 3.80, 95% CI 1.55–9.33) (Table 3). Additionally, participants with FI were over four times more likely to have undergone a previous colonoscopy (OR 4.38, 95% CI 1.40–13.67), over twice as likely to have had a previous appendicectomy (OR 2.21, 95% CI 1.02–4.80) and almost three times more likely to have had a previous cholecystectomy (OR 2.96, 95% CI 1.10–7.99). However, FI was not significantly associated with age, gender or ethnicity. Previous obstetric trauma did not appear to be a significant factor for FI (see Table 3).

Overall, urinary incontinence was reported by one‐quarter of participants, almost all of whom were women (OR 5.01, 95% CI 2.62–9.59) and Caucasian (OR 1.85, 95% CI 1.10–3.14). Notably, participants who were incontinent to urine were over three times more likely to report FI (OR 3.24, 95% CI 1.73–6.08) (see Table 3).

## Discussion

This study used explicit and contemporary standardized criteria and scoring systems to assess the prevalence of FBDs and FI (and its severity) and confirmed that they are common, being present in 33% of Australian primary healthcare seekers. Specifically, the Rome III criteria for IBS were met in 11%, most commonly in Caucasians, with participants being more likely to report anxiety and/or depression and have had a previous colonoscopy or cholecystectomy. Functional constipation was present in 8%, with only 3% of patients using laxative medication. FI was present in 12% of participants (5% to solid stool and 7% to liquid stool) with the majority (52%) reporting moderate/severe incontinence. FI was associated with previous anal and abdominal surgery and concomitant urinary incontinence.

The comparison of prevalence between this and other published studies is hampered by varying diagnostic criteria and study methodologies employed. For example, the published prevalence rates of IBS range from 0.8% to 28% 4. Only one previous study has assessed community prevalence of IBS in a Western population using Rome III criteria, reporting a prevalence of 16% 25. The prevalence of IBS in the present study (11%) was comparable to that reported in a recent community‐based Australian study, which used Rome II criteria and diagnosed IBS in 8.9% of subjects 26. The only previous study focusing on primary healthcare seekers was conducted over a decade ago and used Manning criteria and diagnosed IBS in almost one‐third of subjects 27.

The association between IBS and Caucasian ethnicity demonstrated in the present study has been previously documented 28, although a reason for this racial preponderance remains poorly understood. Female gender and individuals with tertiary education or employment status have previously been shown to be associated with IBS 13, although we found no relationship amongst these factors in the present study. The observed significant association between mental health issues (e.g. depression and anxiety) and IBS has also been well documented 29, although the issue of cause, effect or epiphenomenon remains unresolved and cannot be answered by the present study.

Our study reveals that individuals with IBS were over nine times more likely to have had a previous colonoscopy and over three times more likely to have had a previous cholecystectomy, in keeping with findings from previous studies 30, 31, 32. The association with colonoscopy, in pursuit of an organic basis for symptoms, is perhaps unsurprising. Many investigations are often performed in such subjects with the hope of increasing the certainty associated with the diagnosis of IBS, but for the majority of cases these investigations add little to the overall diagnostic schema 31. Indeed, the use of symptom‐based taxonomies, such as the Rome III criteria, for population‐ and clinic‐based studies is justified by the validity and reliability of the objective measures to diagnose FGIDs based on symptoms alone, without the need for formal investigations 33.

The prevalence of functional constipation has also varied significantly in published studies. For example, a recent systematic review of constipation prevalence in North America reported rates ranging from 1.9% to 27.2%, although the majority of included studies reported rates from 12% to 19% 16. Previous Australian estimates of the prevalence of constipation have varied from 2.8% 26 to 30.7% 34. The prevalence of 8% reported in the present study falls within this wide range from population‐based studies although, to the best of our knowledge, it is the first study to specifically assess its prevalence among primary healthcare seekers.

The prevalence of FI in the literature ranges from 2% in the adult population 35 to 15% in more elderly populations 21, 36. However, rates are much higher in specific groups, with 50% of nursing home residents 37 and patients with multiple sclerosis 38 and 20% of patients with diabetes mellitus 39 reporting FI. Accordingly, the prevalence estimates of FI are influenced by the demographics and characteristics of the population studied. Further, it may be influenced by variation in the definition used for diagnosis and symptom severity assessment. The prevalence of FI measured in this study is remarkably similar to that of a previous Australian population‐based community study, which reported a prevalence of 11.2% 40. The same study reported solid and liquid stool incontinence in 2% and 9% of subjects, respectively, compared with rates of 5% and 7% observed in the present study.

Participants with FI were four times more likely to have had previous anal surgery. Although the timing of surgery in relation to the development of symptoms was not formally explored, anal surgery has been previously recognized as the most important aetiological factor for the development of FI in men 41 due to the injurious effects on anal sphincter function 42. Interestingly, the present study did not find an association between obstetric trauma and FI, despite the relationship being previously documented 20 and presumed secondary to direct sphincter damage and/or pudendal neuropathy 43. Of interest, our findings of a non‐association between obstetric trauma and FI is in agreement with recent large population‐based studies, which instead identified other factors such as age and diarrhoea to be much more relevant in predisposing a subject to FI 44.

Additionally, participants with FI were four and three times more likely to have had a previous colonoscopy and cholecystectomy, respectively. The association with colonoscopy may be related to increased rates of luminal imaging performed in these participants to exclude an organic basis for FI (e.g. inflammatory bowel disease or partially obstructing neoplastic lesions). The association between FI and previous cholecystectomy has only been reported in one previous study 44 with no firm explanation provided. Theoretically, alterations in bile salt metabolism may alter stool consistency sufficiently to challenge the sphincter complex and explain the higher rates of FI in post‐cholecystectomy subjects. The association between FI and previous appendicectomy demonstrated in the present study has never been previously documented and remains unexplained. Finally, the relationship between urinary incontinence and FI is consistent with previous studies 42, 45 and may reflect pelvic floor dysfunction and/or abnormalities of sensorimotor nerve function given that common afferent/efferent nerves from the sacral spinal cord innervate these viscera and their sphincters, and may conceivably be subject to disruption from a common underlying aetiology 46, 47, 48.

Our finding that over one in 10 primary healthcare seekers report FI is of great clinical relevance to primary and secondary care providers alike. This is all the more pertinent given the increasing array of non‐surgical and surgical treatment options currently in the colorectal surgeon's armamentarium to manage FI. In recent years, well‐designed and executed randomized, controlled trials 1 have confirmed the clinical effectiveness of non‐surgical interventions, such as optimization of anti‐diarrhoeal agents, physical supports such as anal plugs, and biofeedback therapy. Surgical interventions include direct sphincter repair, perianal injection of biomaterials and more complex procedures (reserved for highly selective cases) such as dynamic graciloplasty and insertion of an artificial bowel sphincter 49. Over the past decade, much interest has surrounded the use of sacral neuromodulation for the treatment of FI, a procedure widely accepted to carry minimal morbidity but with medium‐term efficacy rates measured to be as high as 81% in a recent systematic review 2.

Further to establishing that FBDs and FI are relatively prevalent in the community, our findings highlight that IBS and constipation remain under‐reported by patients and/or under‐diagnosed (and thus probably under‐treated) by medical practitioners. This is demonstrated by only one‐third of participants meeting the Rome III criteria for IBS having previously been diagnosed with the condition, and that only 3% of participants who met the Rome III criteria for functional constipation were taking regular laxatives. In addition, there are continued challenges in relation to the ‘accurate diagnosis’ of FBDs, since over 50% of participants reporting a previous diagnosis of IBS did not actually meet the objective Rome III criteria at the time of evaluation in this cross‐sectional study.

The association with previous abdominal and pelvic surgery in patients with IBS also warrants discussion. Previously, it has been reported that excessive medical treatment, particularly surgery, has been rendered to sufferers and accounts for the economic burden of IBS estimated at USD$1.6–10 billion in direct costs and USD$19.2 billion in indirect costs 14, 50. It is possible that operative management was (erroneously) performed in some patients with recurrent abdominal pain in the belief that there was an organic basis for the development of recurrent or chronic symptoms (e.g. gallstones or clinical symptoms suggestive of a diagnosis of appendicitis). Unfortunately, the histopathology of resected specimens was not readily available for scrutiny to determine whether organic pathology was identified in these patients. Conversely, the counter‐argument is that such surgery may promote the genesis of functional disorders such as IBS 32. Given the non‐experimental cross‐sectional design of the study, we cannot definitively establish the time order of effects and direction of causation.

The study was limited by the convenience sampling of medical practices in which these surveys were conducted, as practices were selected primarily from general practitioners willing to participate in the study rather than at random. Furthermore, surveys of individuals attending primary care may be subject to selection bias as the study population may represent motivated healthcare seekers and/or the ‘worried‐well’ in varying proportions which may explain the greater proportion of women, senior citizens and overseas migrants included in our sample compared with the New South Wales adult population 51. Additionally, for reasons of patient privacy, the individual reason(s) for seeking primary care was not ascertained, and it remains unknown what proportion of participants presented because of gastrointestinal symptoms. Finally, additional red‐flag symptoms, potentially reflecting underlying organic pathology, were not sought in the study and thus it is possible that the symptoms recorded reflected organic pathology rather than functional disorders in a proportion. However, previous studies have shown that endoscopic and radiological investigations identify organic gastrointestinal lesions in less than 1% of patients who meet symptom‐based criteria for IBS 4, 52, 53. Despite these potential limitations, the universal access of primary healthcare in Australia, consecutive recruitment of participants, response rate (66%) and comparability of our prevalence of FBDs to other community and population‐based studies increase the generalizability and robustness of our findings. The use of validated questions derived from the Rome III criteria for FBDs and Vaizey incontinence score for FI also allowed objective assessment of symptoms and meaningful comparisons with other studies that have used similar criteria.

In conclusion, this study highlights that FBDs and FI are prevalent in primary healthcare seekers, affecting up to one‐third of patients. Given that the population is motivated to seek medical attention, it is probable that sufferers will wish to seek specialist attention should their symptoms deteriorate with time to become sufficiently severe. Further, it is possible that many more could be referred for expert multidisciplinary education and management if primary care providers actively enquired about symptoms and/or were made aware of the specialist services provided by colorectal surgeons.

The study has also demonstrated that these disorders are commonly under‐diagnosed or incorrectly diagnosed and inadequately treated in up to two‐thirds of patients. These findings, and the frequency of complex associations with other symptoms/conditions, further emphasize the need for detailed and accurate assessment of such patients, preferably by expert coloproctologists, to guide appropriate treatment and identify the small subgroup that requires surgical intervention.

## Author contributions

Dr Kheng‐Seong Ng is primary author of the manuscript, and contributed to analysis of data, as well as writing and reviewing the final manuscript. Dr Natasha Nassar contributed to analysis of data, as well as writing and reviewing the final manuscript. Drs Keith Hamd and Asvini Nagarajah were involved in project design and data collection. Professor Marc Gladman was involved in project conception/design and data analysis, as well as writing and reviewing the final manuscript.

## Conflicts of interests

No conflicts of interest exist.

## References

[1] Norton C , Chelvanayagam S , Wilson‐Barnett J , Redfern S , Kamm MA . Randomized controlled trial of biofeedback for fecal incontinence. Gastroenterology 2003; 125: 1320–9. 14598248 10.1016/j.gastro.2003.09.039

[2] Thin NN , Horrocks EJ , Hotouras A *et al* Systematic review of the clinical effectiveness of neuromodulation in the treatment of faecal incontinence. Br J Surg 2013; 100: 1430–47. 24037562 10.1002/bjs.9226

[3] Samaranayake CB , Luo C , Plank AW , Merrie AE , Plank LD , Bissett IP . Systematic review on ventral rectopexy for rectal prolapse and intussusception. Colorectal Dis 2010; 12: 504–12. 19438880 10.1111/j.1463-1318.2009.01934.x

[4] Corazziari E . Definition and epidemiology of functional gastrointestinal disorders. Best Pract Res Clin Gastroenterol 2004; 18: 613–31. 15324703 10.1016/j.bpg.2004.04.012

[5] Drossman DA , Li Z , Andruzzi E *et al* U.S. householder survey of functional gastrointestinal disorders. Prevalence, sociodemography, and health impact. Dig Dis Sci 1993; 38: 1569–80. 8359066 10.1007/BF01303162

[6] Koloski NA , Talley NJ , Boyce PM . Epidemiology and health care seeking in the functional GI disorders: a population‐based study. Am J Gastroenterol 2002; 97: 2290–9. 12358247 10.1111/j.1572-0241.2002.05783.x

[7] Kellow JE . The ‘pro’ case. The Rome III Criteria. Neurogastroenterol Motil 2007; 19: 787–92. 17883429 10.1111/j.1365-2982.2007.01002.x

[8] Quigley EM . The ‘con’ case. The Rome process and functional gastrointestinal disorders: the barbarians are at the gate!. Neurogastroenterol Motil 2007; 19: 793–7. 17883430 10.1111/j.1365-2982.2007.01000.x

[9] Drossman DA . The functional gastrointestinal disorders and the Rome III process. Gastroenterology 2006; 130: 1377–90. 16678553 10.1053/j.gastro.2006.03.008

[10] Longstreth GF , Thompson WG , Chey WD , Houghton LA , Mearin F , Spiller RC . Functional bowel disorders. Gastroenterology 2006; 130: 1480–91. 16678561 10.1053/j.gastro.2005.11.061

[11] Talley NJ . Irritable bowel syndrome: definition, diagnosis and epidemiology. Baillieres Best Pract Res Clin Gastroenterol 1999; 13: 371–84. 10580915 10.1053/bega.1999.0033

[12] Inadomi JM , Fennerty MB , Bjorkman D . Systematic review: the economic impact of irritable bowel syndrome. Aliment Pharmacol Ther 2003; 18: 671–82. 14510740 10.1046/j.1365-2036.2003.t01-1-01736.x

[13] Cremonini F , Talley NJ . Irritable bowel syndrome: epidemiology, natural history, health care seeking and emerging risk factors. Gastroenterol Clin North Am 2005; 34: 189–204. 15862929 10.1016/j.gtc.2005.02.008

[14] Chang L . Review article: epidemiology and quality of life in functional gastrointestinal disorders. Aliment Pharmacol Ther 2004; 20(Suppl 7): 31–9. 10.1111/j.1365-2036.2004.02183.x 15521853

[15] Gschossmann JM , Haag S , Holtmann G . Epidemiological trends of functional gastrointestinal disorders. Dig Dis 2001; 19: 189–94. 11752836 10.1159/000050679

[16] Higgins PD , Johanson JF . Epidemiology of constipation in North America: a systematic review. Am J Gastroenterol 2004; 99: 750–9. 15089911 10.1111/j.1572-0241.2004.04114.x

[17] Agrawal A , Whorwell PJ . Review article: abdominal bloating and distension in functional gastrointestinal disorders – epidemiology and exploration of possible mechanisms. Aliment Pharmacol Ther 2008; 27: 2–10. 17931344 10.1111/j.1365-2036.2007.03549.x

[18] Thompson WG , Irvine EJ , Pare P , Ferrazzi S , Rance L . Functional gastrointestinal disorders in Canada: first population‐based survey using Rome II criteria with suggestions for improving the questionnaire. Dig Dis Sci 2002; 47: 225–35. 11837727 10.1023/a:1013208713670

[19] Bharucha AE , Wald A , Enck P , Rao S . Functional anorectal disorders. Gastroenterology 2006; 130: 1510–8. 16678564 10.1053/j.gastro.2005.11.064

[20] Johanson JF , Lafferty J . Epidemiology of fecal incontinence: the silent affliction. Am J Gastroenterol 1996; 91: 33–6. 8561140

[21] Roberts RO , Jacobsen SJ , Reilly WT , Pemberton JH , Lieber MM , Talley NJ . Prevalence of combined fecal and urinary incontinence: a community‐based study. J Am Geriatr Soc 1999; 47: 837–41. 10404928 10.1111/j.1532-5415.1999.tb03841.x

[22] Walter S , Hallbook O , Gotthard R , Bergmark M , Sjodahl R . A population‐based study on bowel habits in a Swedish community: prevalence of faecal incontinence and constipation. Scand J Gastroenterol 2002; 37: 911–6. 12229965 10.1080/003655202760230865

[23] Department of Human Services . Medicare services, 2013 Available from: http://www.humanservices.gov.au/customer/subjects/medicare-services (accessed 03 July 2013).

[24] Vaizey CJ , Carapeti E , Cahill JA , Kamm MA . Prospective comparison of faecal incontinence grading systems. Gut 1999; 44: 77–80. 9862829 10.1136/gut.44.1.77 PMC1760067

[25] Krogsgaard LR , Engsbro AL , Bytzer P . The epidemiology of irritable bowel syndrome in Denmark. A population‐based survey in adults ≤ 50 years of age. Scand J Gastroenterol 2013; 48: 523–9. 23506174 10.3109/00365521.2013.775328

[26] Boyce PM , Talley NJ , Burke C , Koloski NA . Epidemiology of the functional gastrointestinal disorders diagnosed according to Rome II criteria: an Australian population‐based study. Intern Med J 2006; 36: 28–36. 16409310 10.1111/j.1445-5994.2006.01006.x

[27] Thompson WG , Heaton KW , Smyth GT , Smyth C . Irritable bowel syndrome in general practice: prevalence, characteristics, and referral. Gut 2000; 46: 78–82. 10601059 10.1136/gut.46.1.78 PMC1727778

[28] Dong YY , Zuo XL , Li CQ , Yu YB , Zhao QJ , Li YQ . Prevalence of irritable bowel syndrome in Chinese college and university students assessed using Rome III criteria. World J Gastroenterol 2010; 16: 4221–6. 20806442 10.3748/wjg.v16.i33.4221 PMC2932929

[29] Spiller RC . Irritable bowel syndrome. Br Med Bull 2004; 72: 15–29. 15767561 10.1093/bmb/ldh039

[30] Kennedy TM , Jones RH . Epidemiology of cholecystectomy and irritable bowel syndrome in a UK population. Br J Surg 2000; 87: 1658–63. 11122180 10.1046/j.1365-2168.2000.01596.x

[31] Suleiman S , Sonnenberg A . Cost‐effectiveness of endoscopy in irritable bowel syndrome. Arch Intern Med 2001; 161: 369–75. 11176762 10.1001/archinte.161.3.369

[32] Longstreth GF , Yao JF . Irritable bowel syndrome and surgery: a multivariable analysis. Gastroenterology 2004; 126: 1665–73. 15188159 10.1053/j.gastro.2004.02.020

[33] Ishihara S , Yashima K , Kushiyama Y *et al* Prevalence of organic colonic lesions in patients meeting Rome III criteria for diagnosis of IBS: a prospective multi‐center study utilizing colonoscopy. J Gastroenterol 2012; 47: 1084–90. 22460220 10.1007/s00535-012-0573-4

[34] Howell SC , Quine S , Talley NJ . Low social class is linked to upper gastrointestinal symptoms in an Australian sample of urban adults. Scand J Gastroenterol 2006; 41: 657–66. 16716963 10.1080/00365520500442567

[35] Perry S , Shaw C , McGrother C *et al* Prevalence of faecal incontinence in adults aged 40 years or more living in the community. Gut 2002; 50: 480–4. 11889066 10.1136/gut.50.4.480 PMC1773171

[36] Peet SM , Castleden CM , McGrother CW . Prevalence of urinary and faecal incontinence in hospitals and residential and nursing homes for older people. BMJ 1995; 311: 1063–4. 7580664 10.1136/bmj.311.7012.1063 PMC2551367

[37] Nelson R , Furner S , Jesudason V . Fecal incontinence in Wisconsin nursing homes: prevalence and associations. Dis Colon Rectum 1998; 41: 1226–9. 9788384 10.1007/BF02258218

[38] Hinds JP , Eidelman BH , Wald A . Prevalence of bowel dysfunction in multiple sclerosis. A Population Survey. Gastroenterology 1990; 98: 1538–42. 2338192 10.1016/0016-5085(90)91087-m

[39] Feldman M , Schiller LR . Disorders of gastrointestinal motility associated with diabetes mellitus. Ann Intern Med 1983; 98: 378–84. 6402969 10.7326/0003-4819-98-3-378

[40] Kalantar JS , Howell S , Talley NJ . Prevalence of faecal incontinence and associated risk factors; an underdiagnosed problem in the Australian community? Med J Aust 2002; 176: 54–7. 11936284

[41] Lunniss PJ , Gladman MA , Hetzer FH , Williams NS , Scott SM . Risk factors in acquired faecal incontinence. J R Soc Med 2004; 97: 111–6. 14996955 10.1258/jrsm.97.3.111 PMC1079318

[42] Aitola P , Lehto K , Fonsell R , Huhtala H . Prevalence of faecal incontinence in adults aged 30 years or more in general population. Colorectal Dis 2010; 12: 687–91. 19486087 10.1111/j.1463-1318.2009.01878.x

[43] Nelson R , Norton N , Cautley E , Furner S . Community‐based prevalence of anal incontinence. JAMA 1995; 274: 559–61. 7629985

[44] Bharucha AE , Zinsmeister AR , Locke GR *et al* Risk factors for fecal incontinence: a population‐based study in women. Am J Gastroenterol 2006; 101: 1305–12. 16771954 10.1111/j.1572-0241.2006.00553.x

[45] Rommen K , Schei B , Rydning A , Sultan AH , Morkved S . Prevalence of anal incontinence among Norwegian women: a cross‐sectional study. BMJ Open 2012; 2: pii: e001257; doi: 10.1136/bmjopen‐2012‐001257. 10.1136/bmjopen-2012-001257 PMC4400729 22850167

[46] Edwards NI , Jones D . The prevalence of faecal incontinence in older people living at home. Age Ageing 2001; 30: 503–7. 11742780 10.1093/ageing/30.6.503

[47] Goode PS , Burgio KL , Halli AD *et al* Prevalence and correlates of fecal incontinence in community‐dwelling older adults. J Am Geriatr Soc 2005; 53: 629–35. 15817009 10.1111/j.1532-5415.2005.53211.x

[48] Whitehead WE , Borrud L , Goode PS *et al* Fecal incontinence in US adults: epidemiology and risk factors. Gastroenterology 2009; 137: 512–7, 7.e1–2. 19410574 10.1053/j.gastro.2009.04.054 PMC2748224

[49] Gladman MA , Knowles CH . Surgical treatment of patients with constipation and fecal incontinence. Gastroenterol Clin North Am 2008; 37: 605–25, viii. 18793999 10.1016/j.gtc.2008.06.009

[50] Saito YA , Schoenfeld P , Locke GR 3rd . The epidemiology of irritable bowel syndrome in North America: a systematic review. Am J Gastroenterol 2002; 97: 1910–5. 12190153 10.1111/j.1572-0241.2002.05913.x

[51] Australian Bureau of Statistics . Community Profiles. 2012 Available from: http://www.abs.gov.au/websitedbs/censushome.nsf/home/communityprofiles (accessed 03 July 2013).

[52] Holmes KM , Salter RH . Irritable bowel syndrome − a safe diagnosis? Br Med J (Clin Res Ed) 1982; 285: 1533–4. 10.1136/bmj.285.6354.1533 PMC1500549 6814632

[53] Svendsen JH , Munck LK , Andersen JR . Irritable bowel syndrome − prognosis and diagnostic safety. A 5‐year follow‐up study. Scand J Gastroenterol 1985; 20: 415–8. 4023607 10.3109/00365528509089673

